# Immediate effect of exercises of scapular stabilization on shoulder and forearm muscle activation patterns while playing the violin (New exercises to strengthen musicians—Part II)

**DOI:** 10.3389/fpsyg.2026.1825818

**Published:** 2026-06-30

**Authors:** Céleste Rousseau, Christoff Zalpour, Ju-Yang Chi, Bronwen J. Ackermann

**Affiliations:** 1 Department of Movement and Rehabilitation Science, Faculty of Business, Management, and Social Science, University of Applied Sciences Osnabrück, Osnabrück, Germany; 2 Unité de Recherche UR 20201 REHADAPT, Université Versailles Saint-Quentin – Paris Saclay, Versailles, France; 3 USR 3608 République des Savoirs, École Normale Supérieure, Paris, France; 4 School of Medical Sciences, Faculty of Medicine and Health, University of Sydney, Sydney, NSW, Australia

**Keywords:** electromyography, motor control, musical performance, scapular stabilization, violinist

## Abstract

**Introduction:**

Playing-related musculoskeletal disorders are prevalent in musicians due to the complex and repetitive physical demands imposed over extended durations, often in awkward postures. For violinists, the position of the arm during precise movements requires good scapular control. Indeed, scapular dyskinesis and altered motor control of stabilizer muscles are often associated with pain in violinists. This study aimed to ascertain whether exercises that activated the scapular stabilizing muscles could lead to immediate changes in motor control or altered muscle patterns while playing the violin.

**Methods:**

In this quasi-experimental study, 3 instrument-specific and 3 classic scapular stabilizer exercises were taught to 12 violinists. Scapular and forearm muscle activation patterns were recorded using electromyography during these exercises (in a randomized order) and while playing their instrument before and after the intervention. Post-exercise questions allowed for subjective evaluations.

**Results:**

The exercises successfully activated the scapular stabilizing muscles during task-specific and non-specific bilateral exercises, with a slight superiority observed for task-specific exercises. However, aside from the right lower trapezius, no immediate pre−/post-playing muscle activation pattern differences were observed.

**Conclusion:**

Purpose-designed exercises can recruit appropriate musculature in violinists, but immediate effects on motor control during playing were not observed. Further research should investigate whether muscle activation patterns may change after a longer training period, where strength changes can occur.

## 1 Introduction

Playing-related musculoskeletal disorders (PRMDs) were first described in 1998 (Zaza et al., 1998) as “pain, weakness, numbness, tingling, or other symptoms that interfere with (their) ability to play (their) instrument at the level (they) are accustomed to.” Since then, numerous authors have worked to better understand the causes of these PRMDs and to investigate effective ways to prevent them. Violinists are consistently reported to suffer particularly high incidences of PRMDs in the back and the upper limbs, as highlighted in a recent systematic review of playing-related problems in violinists (Zinn-Kirchner et al., 2023), most commonly affecting the right shoulder and the left forearm (Ackermann et al., 2012; Moraes and Papini, 2012).

Concerning the movements involved in playing the violin, the musician must hold the instrument between the left shoulder and chin (Tawde et al., 2016), thus creating a static muscular load on the left neck and shoulder muscles, including the left upper trapezius, while simultaneously pressing the strings at various places along the fingerboard with their left fingers (Cattarello et al., 2017). With the right upper limb, the violinist moves a bow back and forth across the strings to create vibrations and, hence, sound, requiring variable levels of right arm elevation combined with downward pressure on the bow. This bowing movement involves a large range of motion of the right shoulder joint; therefore, scapular stabilization is important for providing both strength and flexibility to the bowing arm (Kibler and Sciascia, 2009).

Scapulae have crucial roles for the upper arm, as they form the sole connection between the trunk and the upper limb, thereby acting as a key element of the kinetic chain, transferring muscular energy from the core and lower limbs and their large muscles via the scapulae to the upper extremities’ smaller ones (Sciascia and Kibler, 2022). Scapular stabilization is reported to result from the synergic contributions of several muscles, with the middle and lower trapezius and serratus anterior reported to be particularly essential to this stability (Sciascia and Kibler, 2022; Cattarello et al., 2017; Mottram et al., 2009). In a previous study, scapular stabilization has been reported to be often impaired in musicians, including upper-string players (Steinmetz et al., 2010). When scapular stabilization is dysfunctional (which is not considered a proper injury but rather a physical impairment), it is called “scapular dyskinesis”; it may come from a wide number of causes, such as serratus anterior and lower trapezius insufficiency (or SALTI), loss of conscious control, or difficulty/inability to retract the scapulae (Sciascia and Kibler, 2022). Studies have shown positive effects of improving the balanced activation of these muscles around the scapula for shoulder blade stabilization (Worsley et al., 2013), with a focus on motor control (to improve scapular dyskinesis) rather than just muscle strengthening (Sciascia and Kibler, 2022). In musicians, a positive association was found between pain symptoms and a higher prevalence of scapular dyskinesis (Silva et al., 2018). Moreover, it has been demonstrated that the shoulder blade position and, particularly, the ability to control movement around a neutral position (between protraction and retraction), influences not only localized muscular activity but also distal effects on grip strength and forearm muscle activation patterns in that arm (Picco et al., 2010). Previously tested exercise programs for musicians are largely non-specific or generalized to all instrumentalists (Nygaard Andersen et al., 2017; Chan et al., 2014), despite evidence that prolonged asymmetrical postures contribute to variable instrument-specific muscle imbalances and performance-related musculoskeletal disorders.

Finally, considering the violinists’ performance and movements from a contemporary motor control perspective that integrates the individual, the task, and the environment, the proposed exercises were also designed to enhance the coordination of proximal and distal body segments (Sciascia and Kibler, 2022) when playing the violin. The development of these exercises also relied on current theories of motor and sensorimotor planning and learning, conceived as perpetual adaptations that can be reshaped by both internal (sensory) and external (performance) feedback (Kim et al., 2021). In addition, the exercises were designed to avoid the dual-task demands of both playing and concentrating on internal sensations, a situation described as potentially altering performance in a negative manner (Allingham and Wöllner, 2022; Duke et al., 2011).

Therefore, in this study, a series of scapular exercises is proposed to address scapular stabilization impairments observed in these players (Tawde et al., 2016; Steinmetz et al., 2010), hence illustrating the potential for increasing proximal support of playing actions performed by the whole arm. Grounded in theoretically informed rehabilitation principles (Sciascia and Kibler, 2022; Kim et al., 2021), this study aims to compare typical exercises frequently used by physiotherapists (Mottram et al., 2009; Picco et al., 2010) and violin-specific functional exercises, designed to target the movements required to play the violin. Developing and investigating such task-specific exercises could help healthcare practitioners working with musicians to implement clinically applicable solutions grounded in both motor control and biomechanical principles (Hudon et al., 2015).

The aims of the current study are:

1. To investigate shoulder and forearm muscle activation patterns while performing a series of scapular stabilizer exercises (including task-specific designs) and while playing the violin.
2. To observe whether the exercises appropriately activate the target muscles, and whether an observed muscle activity pattern change immediately transfers into a functional outcome within a single clinical visit scenario.

The hypothesis to be tested is that immediate changes in motor control while playing the violin may occur following scapular stability exercises that train changes in muscle activation patterns using sEMG biofeedback.

## 2 Method

The design of this study is quasi-experimental, including tests before and after six conditions, and pre- and post-intervention questionnaires.

### 2.1 Sample description

The sample consisted of 12 participants, 6 women and 6 men, with an average age of 26.9 ± 7.8 years. The average lifetime playing experience was 18.5 ± 8.3 years and 7.2 ± 5.2 years in an orchestra. Practice averaged approximately 12.6 ± 9 h per week. The inclusion criteria for the study were: aged between 18 and 50 years (Kok et al., 2015); able to speak English fluently; and having played the violin for at least 5 years. This study was approved by The University of Sydney Human Research Ethics Committee (HREC no.: 2018/219).

### 2.2 Pre- and post-intervention questionnaires and functional tests

At least two researchers were present for each testing session. After reading a participant information sheet and then signing informed consent, participants were asked to fill in three questionnaires before the intervention: the Musculoskeletal Pain Intensity and Interference Questionnaire for Musicians (MPIIQM, Berque et al., 2014) to investigate the performance-related pain prevalence according to Zaza’s aforementioned definition, the Depression, Anxiety and Stress Scale (DASS-21) to collect psychological data (Adu et al., 2025), and a pre-intervention questionnaire which gathered information on the scapula and physical activity among the sample (Appendix A). In addition, a brief physical examination was conducted by the same researcher: (i) acromion-wall distance and (ii) a scapular dyskinesia test (based on McClure et al., 2009). At the end of the intervention, a post-intervention questionnaire was completed (Appendix B).

### 2.3 Surface electromyography protocol

The muscles selected for sEMG included bilateral scapular and shoulder muscles (upper and lower trapezius, pectoralis major, serratus anterior (Mottram et al., 2009; Maenhout et al., 2016), and anterior deltoid), as well as the common flexor and extensor muscles of the forearm. SENIAM recommendations (Hermens et al., 2000) were used to guide the sEMG protocol for the shoulder muscles, and Criswell (2010) was used for the other electrode placements. Prior to electrode placement, the skin was prepared by vigorously rubbing with alcohol and abrasive gel (NuPrep, Aurora, CO, United States) to reduce impedance. Two Ag/AgCl surface electrodes (Red Dot, 2,258; 3 M, Sydney, NSW, Australia) were placed 2 cm apart in parallel with the muscle fibers of each selected muscle. Fixomull hypoallergenic adhesive tape (Smith & Nephew, North Ryde, NSW, Australia) was applied as necessary to prevent electrode movement during trials. Electrodes were connected to wireless sEMG sensors (TELEmyo DTS EMG sensors; Noraxon, Scottsdale, AZ, United States – ~14 g, 3.4 × 2.4 × 1.4 cm) and amplified with a first-order band-pass filter (bandwidth 10–500 Hz, gain of 500, input impedance >100 MΩ, and common-mode rejection >100 dB). The amplified signals were transmitted to a 16-bit-resolution receiver (TELEmyo DTS belt receiver, Noraxon) and recorded to a computer at 1500 Hz using MR3 software (version 3.6.20; Noraxon). Electrodes were also placed in a playing position to prevent skin sliding.

The same researcher conducted all Maximal Voluntary Contraction (MVC) tests, in which participants were instructed to produce an isometric contraction with maximum effort against resistance for 3 s. Each MVC was repeated three times, separated by a 20-s rest period. The following seven MVCs were performed:

- Flexion and extension of the wrist at 90° elbow flexion, with the forearm supported by a table (Weerakkody et al., 2003).
- Five standardized MVC shoulder exercises (Ginn et al., 2011): shoulder internal rotation at 90° elbow flexion, abduction at 90° abduction, shoulder flexion at 125° flexion, and shoulder extension at 30° abduction.

Signal processing was performed with the Noraxon software. EMG signals were high-pass filtered at 10 Hz (zero-lag, eighth-order Butterworth), rectified, and then the linear envelope was calculated by low-pass filtering at 3 Hz (zero-lag, eighth-order Butterworth). All signals were visually inspected prior to processing by blinded study personnel. The EMG signals in the excerpt were then normalized to the maximal value of the recorded signals and expressed as %MVC.

### 2.4 Exercises and protocol session description

Participants were asked to play three octaves of the C major scale, up and down, at three different standardized tempi: 60 bpm, 120 bpm with the upper half of the bow, and 120 bpm with the lower half of the bow. They were asked to play this task before and after being taught the scapular stabilization exercises. While performing the exercises and playing post-exercise, participants were given verbal prompts to encourage them to feel their scapular muscles and increase their awareness. Before the very first session, the three physiotherapists (B. A., J.-Y. C., and C. R.) who developed the protocol agreed on standardized instructions to keep them consistent across all participants. Two physiotherapists (J.-Y. C. and C. R.) were present at every testing session.

Each participant was taught six exercises, which were assigned at random. Participants then performed each exercise five times (this number of repetitions was previously assessed as adequate for activating the targeted muscles in a pilot study). Of these exercises, three had been purpose-designed and previously tested to best mimic arm-movement-specific actions during violin playing, thereby activating the scapular stabilizing muscles.

The exercises included:

- X1: bilateral rotation with resistance in the play position (Chan et al., 2014).
- X2 (left) and X3 (right): unilateral exercises of scapular stabilization with resistance.

These exercises were developed during a pilot study and are the most relevant for recruiting the scapular stabilizing muscles. In addition, typical scapular stabilizer exercises were performed, which are often used by physiotherapists in shoulder rehabilitation:

- X4: bilateral rotation of the shoulder with resistance (Castelein et al., 2016).
- X5: Scapular Orientation Exercise (Mottram et al., 2009).
- X6: push-up against the wall (Maenhout et al., 2016).

The resistance was performed using a low-resistance band (red TheraBand^®^, United States of America) to more closely mimic the lighter-to-moderate effort used over these prolonged time periods by violinists and violists.

All exercises were explained and demonstrated by the primary researcher (C.R.) to enhance the consistency of the approach used in Table 1. The range of movements was not standardized, as the exercises should mimic as closely as possible the violinists’ movements.

**Table 1:** General and specific exercises of scapular stabilization.

Exercise number	Starting position	Instructions	Picture
X1: Bilateral rotation in play position	Arms placed in the violin play position, Theraband^®^ between both wrists, pen in the bowing hand.	Stretch the elastic by spreading your wrists with a movement of your right upper limb, in position of playing the violin, feeling your shoulder blade muscles and opening your chest.	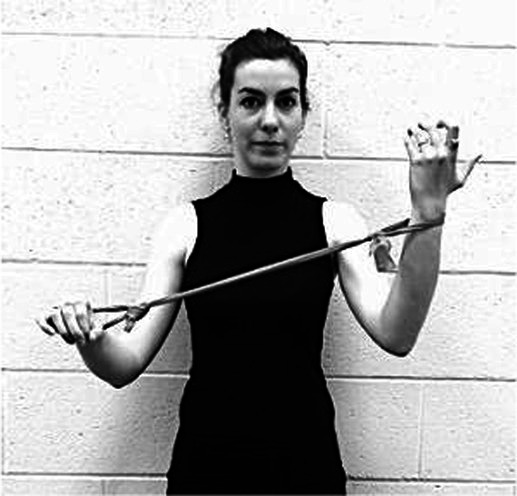
X2: Left exercise of scapular stabilisation	Arms placed in the violin play position, Theraband^®^ around one wrist, fixed on the front, pen in the bowing hand.	Engage your shoulder blade first and then stretch the elastic by spreading your wrist from the wall forward, as if you were playing along the violin neck, with opening your chest and feeling your shoulder blade muscle.	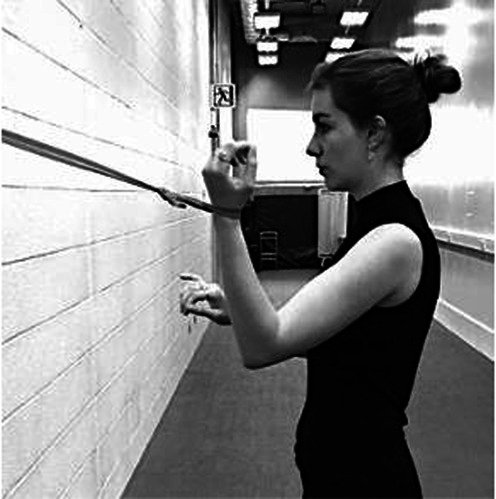
X3: Right exercise of scapular stabilisation	Arms placed in the violin play position, Theraband^®^ around one wrist, fixed on the front, pen in the bowing hand.	Engage your shoulder blade first and then stretch the elastic by spreading your wrist from the wall forward, as if you were bowing, with opening your chest and feeling your shoulder blade muscle. You can also feel your weight moving between your both legs.	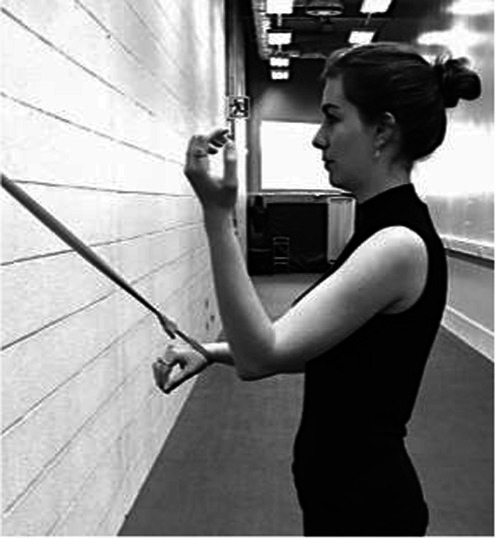
X4: Bilateral external rotation	Shoulder and elbows flexed at 90°, Theraband^®^ held in hands.	Stretch the elastic by spreading your hands, feeling your shoulder blade muscles and opening your chest.	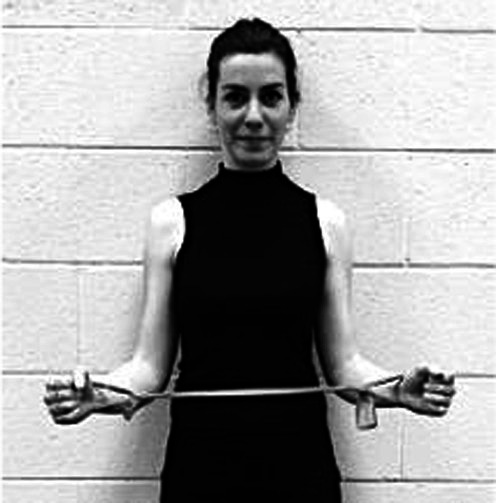
X5: Scapular orientation exercise	Stand, relaxed.	Make your shoulder blades down and closer, with opening your chest and feeling your shoulder blade muscles.	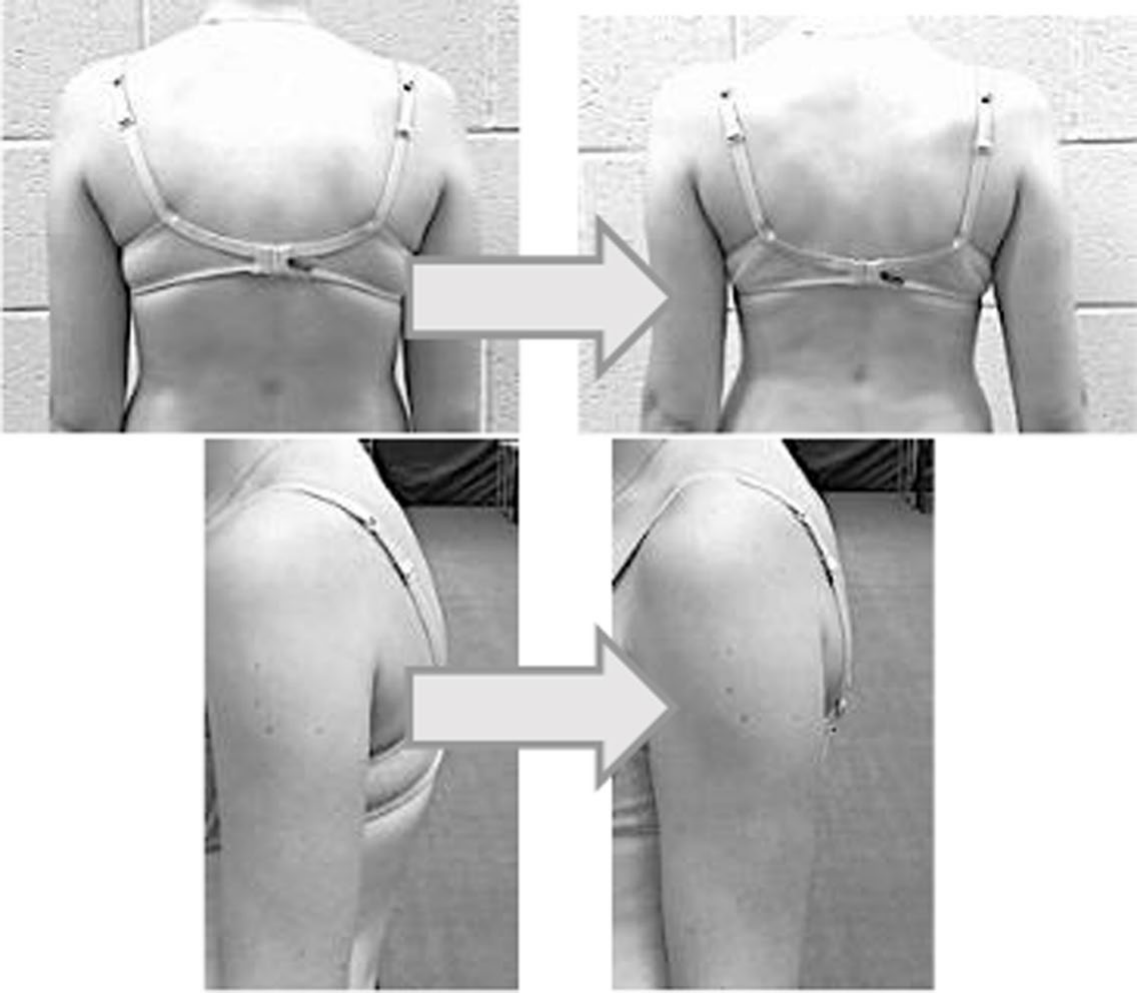
X6: Push-up against the wall	One step backward from the wall, palms against the wall.	Go closer to the wall by make your chest closer and flexing your elbows.	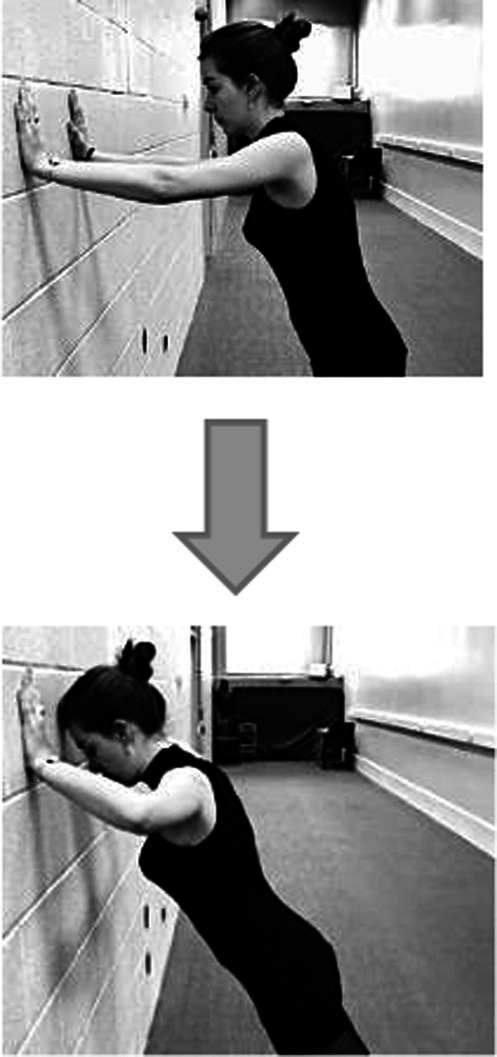

After each exercise, the scales were repeated, and in addition to the sEMG recordings, two questions were asked of the participants using a Likert scale. The participants could choose a score between −5, representing an extremely negative effect on playing, and +5 indicating an extremely positive effect on playing.

The questions were:

- Can you rate how much you felt your shoulder blade muscles during this exercise?
- Can you rate how much this exercise had an impact on the feel of your shoulder blade muscles while playing?

At the end of the intervention, participants were asked, using a post-intervention survey (Appendix B), to evaluate several items about the shoulder blade exercises and their perceived effectiveness, reproducibility, utility, and likelihood of performing them again in the future.

### 2.5 Statistical analysis

All statistical analyses were performed using SPSS (version 1.0.0.950) on macOS X. EMG data were checked and confirmed to be normally distributed using probability plots. During the exercises, a repeated-measures ANOVA (with Bonferroni *post hoc* tests) was conducted on the mean percentages of muscle activation. Concerning the muscle activation while playing, a paired Student’s *t*-test was first conducted between the pre- and post-intervention recordings for each exercise tempo. Then, a repeated-measures ANOVA (with Bonferroni *post hoc* tests) was conducted on the differences between the pre- and post-intervention recordings of the mean percentages of muscle activation. Concerning the survey questions after the second playing task, a repeated-measures ANOVA (with Bonferroni *post hoc* tests) was conducted.

## 3 Results

### 3.1 Pre-intervention questionnaires and function test outcomes

Responses to the MPIIQM revealed that 11 of the 12 violinists (92%) reported a lifetime prevalence of pain; 7 participants (58%) reported pain throughout the past year; 6 participants (50%) over the past month; and 5 participants (42%) over the past week. Concerning physical activity among the participants, 9 participants (75%) reported regular physical activity (2.2 ± 0.9 h per week), with 6 participants reporting moderate intensity (50%) and 3 participants (25%) reporting moderate-to-vigorous intensity.

According to the DASS-21 outcomes, most participants reported normal to moderate stress and anxiety. However, 3 participants (25%) reported severe stress and 4 participants (33%) reported severe anxiety, while none reported severe depression.

When asked about the ideal scapula position, participants showed a limited understanding of this concept. Eight participants (42%) mentioned “relaxed” to describe this position, and two participants mentioned the idea of “movement freedom” (17%). One participant mentioned an absence of protraction, and another reported that the scapulae must be retracted and not too mobile. Three violinists had no idea what an ideal scapular position might be. Four participants (33%) had previously done some kind of scapular stabilization exercises, including two (17%) who had done so under physiotherapist supervision.

For the physical examination, two examiners scored the dumbbell test, with the following results observed:

- During flexion: Five of the twelve participants (42%) had right scapular dyskinesis, and five of the twelve participants (67%) had left scapular dyskinesis.
- During abduction: Three of the twelve participants (25%) had right scapular dyskinesis, and two of the twelve participants (17%) had left scapular dyskinesis.

For the wall test—to evaluate the difference between the distance of the left and right acromion from the wall—the left was always anterior to the right, despite the varying handedness of the players, this difference did not reach statistical significance.

### 3.2 Muscle activation

Regarding muscle activation patterns, significant differences were found among the six scapular stabilization exercises regarding their relative activation effects on the scapular muscles. These differences are shown in Table 2.

**Table 2:** Average of muscle activation during the exercises (%MVC).

Muscles	Exercises					
	X1 %MVC (SD)	X2 %MVC (SD)	X3 %MVC (SD)	X4 %MVC (SD)	X5 %MVC (SD)	X6 %MVC (SD)
Right upper trapezius	4.8 ± 2.8^*X2,5^	1.3 ± 1.0	3.7 ± 2.9	5.6 ± 3.1^*X2,5^	2.1 ± 1.4	10.5 ± 5.3^*X1,2,3,5^
Left upper trapezius	5.2 ± 1.9^*X2,3,5^	3.0 ± 1.6	2.2 ± 1.7	5.3 ± 2.3^*X2,3^	2.6 ± 2.4	9.5 ± 4.1^*all^
Right middle trapezius	12.7 ± 7.3	6.0 ± 4.4	9.1 ± 4.9	13.9 ± 7.6^*X2,3^	12.3 ± 6.3^*2^	7.3 ± 3.8
Left middle trapezius	22.3 ± 16.7	13.8 ± 6.0	9.7 ± 4.9	19.6 ± 8.3^*X3^	20.8 ± 14.6^*X6^	10.3 ± 7.1
Right lower trapezius	13.2 ± 4.2^*X2,6^	8.6 ± 5.0	12.9 ± 5.7 ^*X2,6^	21.8 ± 8.6^*all^	14.6 ± 7.4^*X2,6^	8.2 ± 5.6
Left lower trapezius	25.3 ± 12.4^*X2,3,5,6^	19.2 ± 10.3^*X6^	13.3 ± 7.8	28.6 ± 13.9^*X2,3,5,6^	16.2 ± 10.0^*X6^	8.4 ± 4.7
Right serratus anterior	5.9 ± 2.4	0.7 ± 0.8	5.6 ± 3.9	6.7 ± 2.6^*X1,2^	0.6 ± 0.5	12.0 ± 3.9^*all^
Left serratus anterior	9.4 ± 4.6^*X1^	7.3 ± 2.4	4.7 ± 5.3	7.5 ± 2.5^*X2,3^	0.7 ± 0.6	11.6 ± 2.2^*all^

X1: Bilateral shoulder rotation in play position; X2: Left scapular control in play position; X3: Right scapular control in play position; X4: Bilateral shoulder rotation; X5: Scapular Orientation Exercise; X6: Push-up against the wall. * Significantly greater than [Xn] with *p* < 0.05.

A repeated-measures ANOVA on the pre-play values demonstrated homogeneity.

Paired Student’s *t*-test revealed the following significant differences:

- Lower activation for the left upper trapezius for X3 at 60 bpm (*t* = 3.595, *p* < 0.05) and 120 bpm (*t* = 3.201, *p* < 0.008) with the upper half of the bow and for X5 at 60 bpm (*t* = 2.221, *p* < 0.05) and 120 bpm with the upper half of the bow (*t* = 2.355, *p* < 0.05).
- Higher activation for the left middle trapezius for X2 at 60 bpm (*t* = −2.567, *p* < 0.05) and 120 bpm with the lower half of the bow (*t* = −3.393, *p* < 0.001).
- Higher activation for the right lower trapezius for X2 at 60 bpm (*t* = −2.579, *p* < 0.05) and lower activation for X4 at 120 bpm (lower half of the bow: *t* = 2.933, *p* < 0.05).
- Higher activation for the left lower trapezius for X3 at 60 bpm (*t* = −2.379, *p* < 0.05).
- Higher activation for the left serratus anterior for X6 at 120 bpm (upper half: *t* = −3.183, *p* < 0.05; lower half: *t* = −2.284, *p* < 0.05).

Repeated-measures ANOVA showed no significant differences between the pre- and post-playing test conditions across all muscles except for the right lower trapezius. Results are shown in Table 3. No significant differences were observed between pre- and post-playing conditions for the forearm common flexor or extensor muscle groups.

**Table 3:** Average of right lower trapezius activation while playing the violin (%MVC).

Muscles	Exercises						
	Tempo	X1 %MVC (SD)	X2 %MVC (SD)	X3 %MVC (SD)	X4 %MVC (SD)	X5 %MVC (SD)	X6 %MVC (SD)
Right lower trapezius	60 bpm	0.3 (± 1.5)	0.7 ± 0.9^*X4^	1.6 ± 3.3^*X4^	−0.7 ± 1.4	0.2 ± 1.3	0.3 ± 1.6
	120 bpm (bow’s upper half)	0.5 ± 1.2^*X4^	0 ± 0.7	1.4 ± 2.5^*X4^	−0.4 ± 1.0	0.3 ± 1.2	0.4 ± 1.5
	120 bpm (bow’s lower half)	0.6 ± 2.7	0.1 ± 1.1	−0.1 ± 2.3	−0.4 ± 0.5	−0.1 ± 0.9	0.2 ± 1.6^*X4^

X1: Bilateral shoulder rotation in play position; X2: Left scapular control in play position; X3: Right scapular control in play position; X4: Bilateral shoulder rotation; X5: Scapular Orientation Exercise; X6: Push-up against the wall. * Significantly greater than [Xn] with *p* < 0.05. MVC, maximal voluntary contraction; SD, standard deviation.

### 3.3 Post-test questionnaire

Regarding the subjective evaluation of the awareness of scapular muscle activation, the task-specific bilateral exercise (3.8 ± 0.9) and the scapular orientation exercise (3.4 ± 1.2) were rated highest; no significant differences were found overall among the six scapular stabilization exercises (Table 4). In the post-test questionnaire, participants reported an immediate positive effect of the exercises on playing (3.3/5 ± 0.9) and on their playing posture (3.4/5 ± 1.2). They estimated their own capacity to reproduce the exercises at 7.1/10 ± 2.0 and their motivation to continue these exercises daily at 7.8/10. They rated the efficiency of adding scapular stabilization while playing at 2.8/5 ± 1.5. Importantly, one participant also mentioned a better sound quality following the exercises.

**Table 4:** Subjective evaluation (Likert scale −5 to +5) of awareness of scapular stabilizer muscles.

Exercises	X1	X2	X3	X4	X5	X6	*p*
During the exercise	3.8 ± 0.9	3.1 ± 1.3	2.7 ± 1.9	3.1 ± 1.0	3.4 ± 1.2	2.8 ± 2.1	0.365
While playing	3.3 ± 1.1	2.1 ± 0.9	2.3 ± 2.4	2.8 ± 1.1	3.1 ± 1.0	2.1 ± 1.8	0.114

X1: Bilateral shoulder rotation in play position; X2: Left scapular control in play position; X3: Right scapular control in play position X4: Bilateral shoulder rotation X5: Scapular Orientation Exercise X6: Push-up against the wall MVC: Maximal voluntary contraction; SD, standard deviation.

## 4 Discussion

The main aim of this study was to investigate how both typical and task-specific exercises for scapular stabilizers might affect the activation of these muscles during violin playing. The results indicate that even with effective recruitment of the scapular stabilizing muscles during purpose-designed scapular stabilization exercises, immediate motor control changes in playing the instrument are not observed.

Using sEMG during the exercises, it was observed that the most effective exercises to recruit the trapezius muscles were bilateral shoulder rotation exercises (task-specific or non-task-specific), whereas push-ups activated the serratus anterior more. Participants reported greater awareness of scapular muscle activation during the scapular orientation (X5) and the task-specific bilateral exercises (X1). Even though the sample for this study was relatively small, muscle activity patterns still showed substantive increases across all exercises, supporting the conclusion that these exercises had the desired effect on the target musculature.

In relation to the immediate effect of the exercises on playing the scales for the second time, we observed a significant increase only in the right lower trapezius at 60 and 120 bpm (upper half of the bow). No significant difference was found regarding the other shoulder or forearm muscles while playing the violin. Given that the participants performed the exercises only five times (for 5 s on average), this change may indicate the possible utility of such functional instrument-specific exercises. However, further studies need to be conducted to investigate which exercises and what duration are required to improve scapular motor control when playing among musicians.

The high prevalence of pain in our sample was comparable to international findings (Ackermann et al., 2012). Regarding the main location of painful symptoms, our results are consistent with other literature (Tawde et al., 2016), with most PRMDs occurring in the shoulders. General psychological scores via the DASS-21 showed that 25% of the participants reported severe stress, and 33% reported severe anxiety.

Concerning physical activity, it is known that playing music involves high muscle activity; some pieces can last more than 3 h and require repetitive gestures. In our participant sample, 25% of the violinists were physically active according to the WHO guidelines (World Health Organization, 2010), whereas 25% were physically inactive. This observation is also consistent with the literature (Stanhope, 2016). It appears that it is still necessary to spread information concerning the importance of daily physical activity and its benefits for physical functioning among the musician population. In addition to this information, the pre-intervention questionnaire revealed that these musicians show a lack of knowledge about their own bodies and their proper functioning, including the function of the shoulder muscles during playing. One likely explanation is that the musicians did not receive any education in basic anatomy or physiology. Educational initiatives are emerging in musicians’ health globally (Chan et al., 2014), but in this sample, health awareness appeared to be low.

Scapular dyskinesis was observed in the participants during the physical examination, particularly during the flexion task, and more so on the left side, which places greater demands on static instrument holding than the right side. This appears consistent with the asymmetrical postural loads associated with the playing position, in which both arms are held in constantly elevated positions, possibly leading to imbalances in the scapula stabilizer muscles.

In addition, a consistently greater distance between the acromion and wall on the left side than on the right side was observed for all participants. These results were not associated with handedness and may be due to the upper string players’ posture, which requires constant elevation and protraction of the left shoulder to hold the violin.

The results of this study concur with previous research indicating that upper string players commonly suffer from playing-related disorders (Tawde et al., 2016). This study also indicates that purpose-designed exercises can immediately activate the desired scapular musculature, as measured via sEMG monitoring. Further studies may indicate the potential role of biofeedback in further assisting such muscle recruitment. However, by demonstrating in this pilot study that these effects do not immediately transfer from exercises into playing actions, violinists and clinicians should not expect immediate functional effects. Further research should aim to ascertain whether the balance of muscle activity around the shoulder changes over a longer-term training program with these exercises, allowing time for strength changes. The McClure scapular dyskinesis test protocol seems useful as part of the assessment–re-assessment process (McClure et al., 2009).

All exercises are literature-based or purpose-designed to be task-specific and based on instrument gestures with sEMG to confirm activation—a completely new innovation. They were designed to be highly practical and simple to teach by physiotherapists to musicians, as evidenced by the immediate activation effects in these muscles observed by monitoring the sEMG. Further research could indicate whether using these exercises regularly over a longer period could help protect the shoulders of violinists from developing PRMDs and, perhaps, based on their feedback, also improve their musical performance. Future studies should include recording each performance to be further judged by external blinded experts to assess if better scapular motor control could also help improve musical performance. Eventually, as this research concentrates on immediate motor learning and control, further studies could also include concepts of implicit motor learning. In this present study, the participants were explicitly asked to recruit and feel their shoulder blade muscles, and to open their chest. Some studies have demonstrated interest in motor learning strategies incorporating an external focus of attention, such as incorporating analogies, metaphors, or environmental constraints to immediately change strategies (Kleynen et al., 2019). Even if playing the violin is a complex task, it could be worthwhile to investigate utilizing these strategies and instructions with musicians to assess whether a quicker translation of functional changes in muscle use occurs. While our hypothesis was not supported—that using biofeedback during scapula activation exercises may lead to immediate transferable changes in muscle activation patterns during violin playing—future research should examine whether the above modifications to the current study protocol may lead to detectable changes.

### 4.1 Strengths and limitations

This exploratory study has several strengths. To our knowledge, it is the first study to investigate the effects of task-specific exercises tailored for violinists on muscle activation. The exercises tested were designed to be easy to implement in clinical practice and simple for physiotherapists and other healthcare practitioners to teach. Moreover, the protocol combined quantitative measurements (e.g., muscle activation via sEMG and various shoulder tests) with subjective data collection. This approach not only helped to better understand the potential effects of the exercises but also revealed that participants considered the task-specific exercises valuable for their playing, even though muscle activation remained unchanged.

However, the study’s findings showed no significant effects on muscle activation or perception among violinists, which may be explained by several limitations. As this was a pilot study, there was a relatively small sample size of participants with highly heterogeneous characteristics, such as pain symptoms, the presence of scapular dyskinesis, or physical activity levels, which were investigated using a non-validated questionnaire. The number of repetitions used was chosen based on the limited literature on this topic and may have been too few to allow enough scapular motor control adaptations to modify playing actions among these expert musicians. Furthermore, because of its short-term duration, the study only investigated immediate flow-over effects, and it is likely that this time was insufficient to create enough changes in well-established motor patterns.

Moreover, scapular motor control is difficult to learn and gain partly because of its body location: while visual feedback is commonly used in motor control, it cannot be easily applied to the scapula (Sciascia and Kibler, 2022). Although one researcher monitored sEMG for effective muscle activity readings during the tasks, it may be more effective for players to use this biofeedback tool themselves to improve the learning of this new scapular motor control (Huang et al., 2013). This can be used in combination with kinematic prompts (Antunes et al., 2016), as both feedback methods have demonstrated effectiveness in increasing participants’ ability to select which muscles to activate or to control their shoulder blades during scapular-focused exercises. Considering the statistical analysis, it would have been valuable to include specific correction methods for the Student’s *t*-test, as it was performed with Bonferroni *post hoc* tests for the ANOVAs.

Finally, when instrumentalists were instructed to focus on their shoulder blade muscle activation, this constituted an “internal focus of attention,” whereby concentration is on muscle activation and the movement itself, in contrast to an “external focus of attention,” or focusing on the outcome of the movement. In a previous study with violinists, a “proximal” external focus of attention, focusing on the resistance of the bow against the string, demonstrated interesting results such as slight and immediate improvement in motor control around the triceps muscle and reduced negative physical thoughts while playing (Allingham and Wöllner, 2022). Combining foci of attention in future studies may also be valuable for helping musicians to better integrate efficient motor control patterns. This could also be combined with proprioceptive training to address a potential loss of proprioception or neuromuscular control, or improve proprioception by performing repetitive and conscious specific movements (Laskowski et al., 1997). While the exercises were evaluated individually for efficacy, they could be combined into an integrated exercise program.

### 4.2 Practical implications

The purpose-designed exercises developed in this study offer specific benefits for violinists’ rehabilitation, as they target the relevant muscles (middle and lower trapezius and serratus anterior) and were deemed valuable by the participants, who recognized the importance of task-specific exercises to further enhance muscle activation while playing. Clinicians should consider assisting musicians in applying these exercises across more than one session.

## 5 Conclusion

Purpose-designed scapular activation exercises for violinists are practical to teach and effectively activate the correct musculature. Results also indicate that immediate motor control effects of these exercises on violin playing should not be anticipated, and further research is needed to determine whether changes in the balance of the scapular stabilization muscles may occur over time. This study aimed to identify potentially useful scapular stabilization exercises applied to the instrument-specific range of motion but did not account for pain or other underlying abnormalities. Future research with larger sample sizes may help determine the relative dosage or utility of these exercises in heterogeneous musician populations.

## Data Availability

The data analyzed in this study can be made available on request. Requests to access these datasets should be directed to celesterousseau.kine@gmail.com.
