# Supplementary figures and images for: Immediate effect of exercises of scapular stabilization on shoulder and forearm muscle activation patterns while playing the violin (New exercises to strengthen musicians—Part II)
Source: Front Psychol. 2026 Jun 30;17:1825818. doi: 10.3389/fpsyg.2026.1825818 (PMC13371711; doi:10.3389/fpsyg.2026.1825818)

Appendix A: Pre-intervention questionnaire


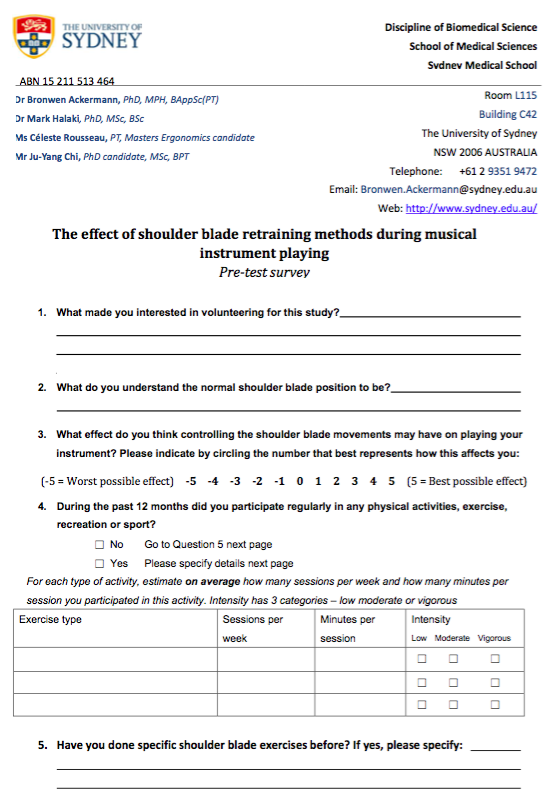


Appendix B: Post-intervention questionnaire


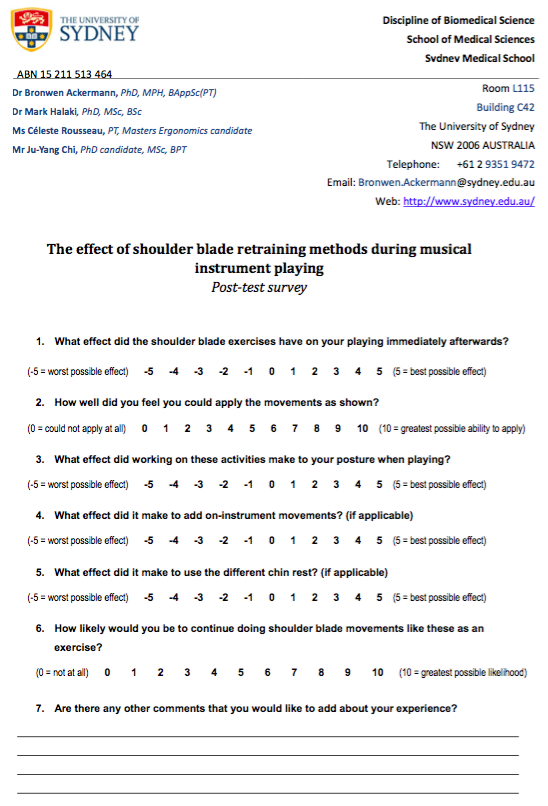

Supplement: Supplementary file 1 [file Supplementary_file_1.docx]
